# Heterogenous antibody and T‐cell responses to SARS‐CoV‐2 mRNA vaccines among immunocompromised young people

**DOI:** 10.1002/ctm2.1183

**Published:** 2023-01-19

**Authors:** Liangjian Lu, Chang Yien Chan, Pauline P. L. Chan‐Ng, Mya Than, Pamela S. Y. Tan, Lee Kean Lim, Sharon Teo, Perry YW Lau, Kar Hui Ng, Elizabeth Y. Ang, Sivaramakrishnan Venkatesh Karthik, Marion M. Aw, Paul A. Tambyah, Hui Kim Yap, Bee Wah Lee

**Affiliations:** ^1^ Department of Paediatrics Khoo Teck Puat – National University Children's Medical Institute National University Health System Singapore Singapore; ^2^ Department of Paediatrics Yong Loo Lin School of Medicine, National University of Singapore Singapore Singapore; ^3^ Division of Infectious Diseases, National University Health System and Department of Medicine Yong Loo Lin School of Medicine, National University of Singapore Singapore Singapore

Dear Editor,

In a representative cohort of immunocompromised young people, we observed marked differences in humoral and cellular responses to a standard two‐dose mRNA vaccination regime between disease categories. These differences are only partially explained by prevailing immunosuppression, which highlights the limitations of the current approach in selecting young patients for additional vaccine doses based primarily on the use of immunosuppressive medication.

Immunocompromised adults are recommended to receive a third primary dose of SARS‐CoV‐2 mRNA vaccines to promote seroconversion, and more recently a second booster dose.^1^, ^2^ There is, however, a paucity of vaccine response data from immunocompromised young people to justify this approach. We characterised the humoral and cellular responses to a standard two‐dose regime of SARS‐CoV‐2 mRNA vaccines in a single‐centre cohort of immunocompromised young people with a representative range of medical conditions and subsequently identified risk factors for an attenuated response.

A total of 97 immunocompromised patients and 22 controls were prospectively recruited. Six participants did not complete the study (Figure S1) and were excluded. The immunocompromised cohort included patients with inflammatory bowel disease (IBD), kidney transplant (KTX), liver transplant (LTX), idiopathic nephrotic syndrome (INS), kidney failure (ESKD), IgA nephropathy/vasculitis (IGA), juvenile idiopathic arthritis (JIA), systemic lupus erythematosus (SLE) and idiopathic uveitis (UVI).

Baseline characteristics (Table 1, Figure 1A) were comparable between controls and the immunocompromised cohort, and disease groups were homogenous in terms of age and dose interval (*p* > .05) which have been reported to affect antibody titres.

**TABLE 1:** Baseline characteristics, immunosuppressive medication use, humoral and cellular vaccine responses in controls and immunocompromised young people

	Controls	All immunocompromised	IBD	LTX	KTX	INS	ESKD	IGA	JIA	SLE	UVI	Others
*N*	20	93	12	8	12	12	11	7	8	12	4	7
**Baseline characteristics^a^**												
Age (years)	17.10 ± .66	17.58 ± .35	16.71 ± .89	17.28 ± 1.35	18.55 ± 1.03	17.43 ± .99	17.82 ± 1.12	18.88 ± 1.51	15.72 ± .9	18.50 ± .93	16.91 ± 1.43	17.29 ± 1.29
Female	7/20 (35)	47/93 (51)	8/12 (67)	5/8 (63)	6/12 (50)	4/12 (33)	1/11 (9)	5/7 (71)	3/8 (38)	11/12 (92)	2/4 (50)	2/7 (29)
Pfizer	18/20 (90)	91/93 (98)	11/12 (92)	7/8 (89)	12/12 (100)	12/12 (100)	11/11 (100)	7/7 (100)	8/8 (100)	12/12 (100)	4/4 (100)	7/7 (100)
Days between doses 1 and 2	31.70 ± 1.75	35.28 ± .93	32.67 ± 1.51	41.63 ± 4.30	39.33 ± 1.48	33.25 ± 2.07	41.09 ± 2.00	30.71 ± 4.3	38.63 ± 3.22	27.33 ± 1.17	32.50 ± 4.33	35.86 ± 4.69
Days after dose 2	38.75 ± 3.22	39.42 ± 1.36	40.92 ± 3.69	46.13 ± 3.36	37.67 ± 3.19	36.08 ± 3.38	38.82 ± 4.12	34.86 ± 3.53	37.63 ± 5.92	38.00 ± 3.75	42.50 ± 8.26	46.14 ± 7.40
**Immunosuppressive medications**												
Steroids	0/20 (0)	26/93 (28)^**^	0/12 (0)	2/8 (25)	12/12 (100)	1/12 (8)	0/11 (0)	4/7 (58)	0/8 (0)	4/12 (33)	0/4 (0)	3/7 (43)
Anti‐metabolites	0/20 (0)	64/93 (69)^***^	11/12 (92)	3/8 (38)	10/12 (83)	11/12 (92)	0/11 (0)	7/7 (100)	4/8 (50)	12/12 (100)	2/4 (50)	4/7 (57)
Calcineurin inhibitors	0/20 (0)	28/93 (30)^**^	0/12 (0)	7/8 (88)	12/12 (100)	5/12 (42)	0/11 (0)	1/7 (14)	0/8 (0)	0/12 (0)	0/4 (0)	3/7 (43)
Biologics	0/20 (0)	16/93 (17)	4/12 (33)	0/8 (0)	0/12 (0)	0/12 (0)	0/11 (0)	0/7 (0)	6/8 (75)	0/12 (0)	3/4 (75)	3/7 (43)
**Humoral response to vaccination**												
Anti‐S > 250 U/ml	20/20 (100)	68/93 (73)^**^	10/12 (83)	8/8 (100)	6/12 (50)	5/12 (42)	11/11 (100)	2/7 (29)	7/8 (88)	8/12 (67)	4/4 (100)	7/7 (100)
Anti‐S > 100 U/ml	20/20 (100)	74/93 (80)^*^	12/12 (100)	8/8 (100)	9/12 (75)	5/12 (42)	11/11 (100)	2/7 (29)	7/8 (88)	9/12 (75)	4/4 (100)	7/7 (100)
Anti‐S > .8 U/ml	20/20 (100)	86/93 (92)	12/12 (100)	8/8 (100)	10/12 (83)	10/12 (83)	11/11 (100)	5/7 (71)	8/8 (100)	11/12 (92)	4/4 (100)	7/7 (100)
**Cellular response to vaccination**												
Pan‐T‐cell IFNγ response (IU/ml)	1.75 (1.43–2.12)	.71 (.59–.87)^*^	.55 (.29–1.06)	.45 (.17–1.24)	.17 (.09–.33)	.53 (.34–.82)	1.54 (.99–2.38)	.75 (.46–1.23)	1.72 (1.20–2.46)	1.99 (1.30–3.05)	.58 (.32–1.03)	.70 (.41–1.21)
Pan‐T‐cell responder	19/20 (95)	66/93 (71)^*^	10/12 (83)	6/8 (75)	5/12 (42)	8/12 (67)	8/11 (73)	5/7 (71)	7/8 (88)	11/12 (92)	2/4 (50)	4/7 (57)
CD4+ T‐cell IFNγ response (IU/ml)	.99 (.81–1.22)	.32 (.25–.42)^*^	.2 (.08–.48)	.28 (.11–.73)	.04 (.02–.10)	.17 (.08–.40)	.89 (.57–1.37)	.4 (.24–.66)	1.3 (.86–1.98)	.84 (.42–1.70)	.35 (.23–.53)	.44 (.21–.92)
CD4+ T‐cell responder	19/20 (95)	72/93 (77)	9/12 (75)	6/8 (75)	4/12 (33)	9/12 (75)	11/11 (100)	6/7 (86)	7/8 (88)	11/12 (92)	4/4 (100)	5/7 (71)

*Note*: ‘Others’ consist of participants with atopic dermatitis (*N* = 2), corneal transplants (*N* = 2) as well as juvenile spondyloarthropathy, juvenile dermatomyositis and membranous glomerulopathy. Biologics used include Adalimumab (*N* = 10), Infliximab (*N* = 2), Tocilizumab (*N* = 2) and Dupilumab (*N* = 2).

Abbreviations: ESKD, end‐stage kidney disease; IBD, inflammatory bowel disease; IGA, IgA nephropathy and IgA vasculitis; INS, idiopathic nephrotic syndrome; JIA, juvenile idiopathic arthritis; KTX, kidney transplant; LTX, liver transplant; SLE, systemic lupus erythematosus; UVI, idiopathic uveitis.

^a^ Frequency data are given as *N*/*N* (%). Summary data are given as mean ± SEM, except for IFNγ responses for which the geometric mean (SEM) is provided.

*, **, ***Refer to significant differences between controls and the immunocompromised cohort with *p* < .05, .01 and .001, respectively.

**FIGURE 1:**
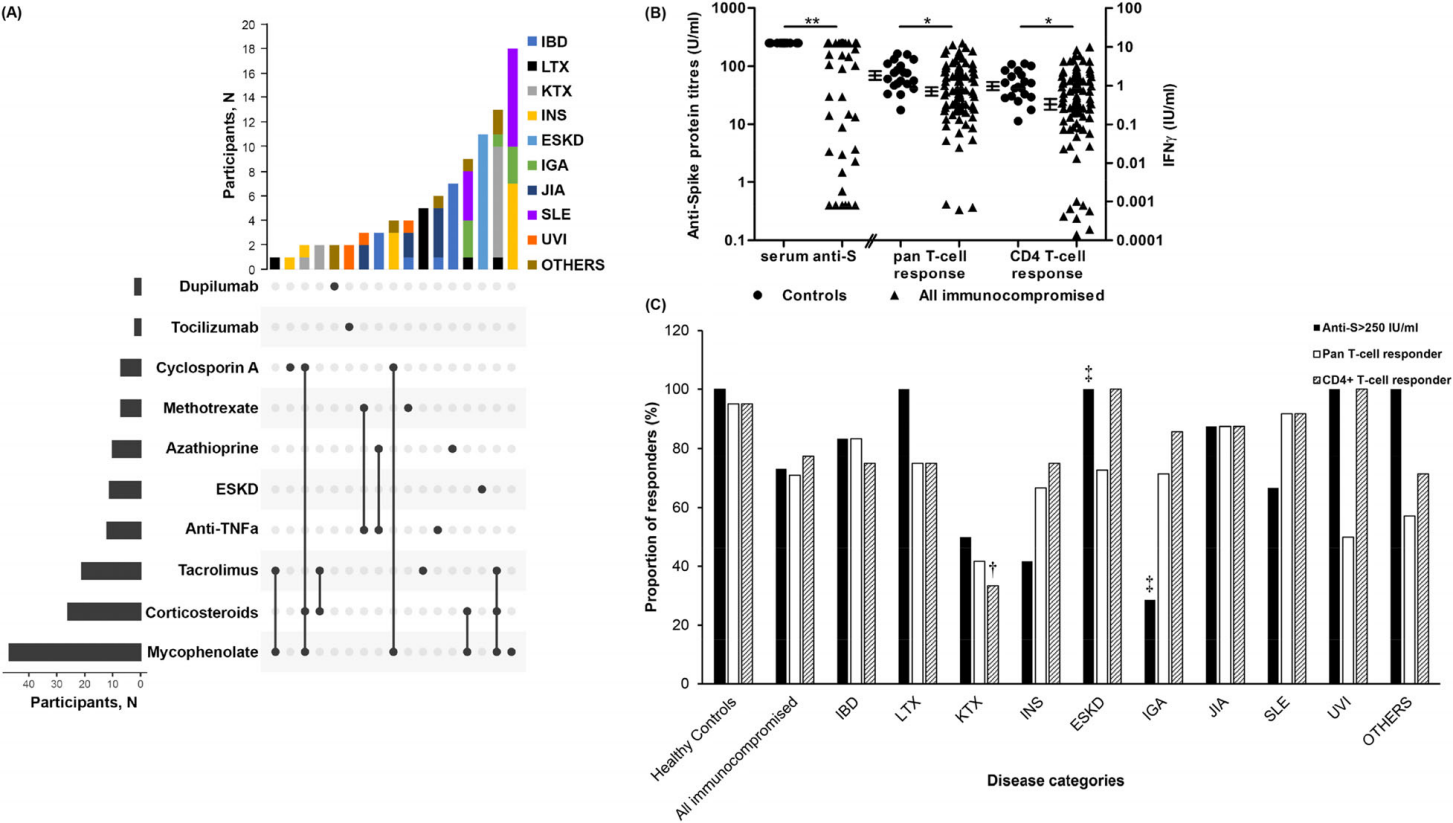
(A) Immunosuppression of immunocompromised participants at time of vaccination. Humoral and cellular responses to vaccination comparing (B) between controls and immunocompromised participants, and (C) within immunocompromised participants across disease categories. *, ** Refer to significant differences between controls and the immunocompromised cohort with *p* < .05 and .01, respectively. ^†^, ^‡^ Refer to BH *p* = .0009 and *p* = .042, respectively, comparing between immunocompromised disease groups. Error bars are geometric mean (SEM).

Following vaccination, only 68 (73%) patients had anti‐spike (Anti‐S) protein titres >250 U/ml, compared to 20 (100%) controls (*p* = .006) (Table 1, Figure 1B). Similarly, patients had reduced pan‐T‐cell (.71 [.59–.87] vs. 1.75 [1.43–2.12] IU/ml, *p* = .041) and CD4+ T‐cell responses (.32 [.25–.42] vs. .99 [.81–1.22] IU/ml, *p* = .048) to SARS‐CoV‐2 spike protein (Table 1, Figure 1B).

Using a definition of non‐responders as those with T‐cell responses two standard deviations below the mean for controls, patients had a lower proportion of pan‐T‐cell responders compared to controls (71% vs. 95%, *p* = .023), but no significant difference in CD4+ T‐cell responders (77% vs. 95%, *p* = .12).

Within immunocompromised patients, there were significant differences between disease groups in the proportion of patients with titers >250 U/ml (*p* < .001) (Table 1, Figures 1C and S2). Compared to immunocompromised patients as a whole, ESKD patients had a superior humoral response with all having titers >250 U/ml (Benjamini–Hochberg [BH] *p*‐value = .042), whereas IGA patients had a poorer humoral response with 29% having titers >250 U/ml (BH *p*‐value = .042). There were also significant differences between disease categories in terms of CD4+ T‐cell responders (*p* = .017) but not pan‐T‐cell responders (*p* = .266). Specifically, the proportion of CD4+ T‐cell responders in KTX patients was reduced compared to immunocompromised patients taken together (BH *p*‐value = .0009).

To examine if the differences in vaccine responses could be explained by the different immunosuppressive medications, we performed a multivariable analysis (Table 2). Patients using corticosteroids (OR = .142 [95% CI: .033–.620], *p* = .009) and anti‐metabolites (OR = .083 [95% CI: .009–.781], *p* = .030) were less likely to have anti‐S titers >250 U/ml, whereas patients using calcineurin inhibitors had reduced pan‐T‐cell (−.641 [95% CI: −1.058 to −.223] log IU/ml, *p* = .003) and CD4+ T‐cell responses (‐.742 [95% CI: −1.290 to −.194] log IU/ml, *p* = .009).

**TABLE 2:** Multivariable analysis of clinical risk factors affecting vaccine responses

	Anti‐S > 250 U/ml			Pan‐T‐cell IFNγ response (log IU/ml)			CD4+ T‐cell IFNγ response (log IU/ml)		
	Odds ratio	95% CI	*p*‐Value	Regression coefficient	95% CI	*p*‐Value	Regression coefficient	95% CI	*p*‐Value
Female	1.878	.528–6.678	.330	−.183	−.537 to .171	.306	−.265	−.73 to .199	.259
Pfizer	0		.999	−.025	−1.263 to 1.213	.968	.133	−1.492 to 1.759	.871
Age (years)	.944	.800–1.114	.497	−.042	−.091 to .008	.101	−.047	−.112 to .019	.158
Days between doses	1.035	.957–1.119	.389	−.003	−.025 to .018	.762	.011	−.017 to .04	.440
Days from dose 2	1.020	.978–1.064	.354	−.005	−.018 to .007	.393	−.007	−.023 to .009	.377
Corticosteroids	.142	.033–.620	.009^**^	−.148	−.601 to .306	.519	−.385	−.98 to .211	.203
Anti‐metabolites	.083	.009–.781	.030^*^	.010	−.42 to .441	.962	.185	−.381 to .75	.518
Calcineurin inhibitors	.861	.204–3.634	.839	−.641	−1.058 to −.223	.003^**^	−.742	−1.29 to −.194	.009^**^
Biologics	.718	.108–4.786	.732	−.367	−.836 to .103	.124	−.284	−.9 to .333	.362

*, **Refer to significant differences between controls and the immunocompromised cohort with *p* < .05 and .01, respectively.

To determine if there was a residual effect of disease category, as disease category and immunosuppressive medications could not be included in the same statistical model due to collinearity (Table S1), we performed a stratified analysis on 28 participants only on anti‐metabolite monotherapy (Table 3). There remained significant differences in pan‐T‐cell responses between categories (*p* = .036), with post hoc testing revealing lower responses in IBD patients compared to SLE patients (.767 [.516, 1.141] vs. 3.97 [2.878–5.478] IU/ml, *p* = .039). There were no significant differences in CD4+ T‐cell (*p* = .093) or antibody (*p* = .214) responses between categories.

**TABLE 3:** Humoral and cellular responses to vaccination amongst participants on anti‐metabolite monotherapy

	IBD	INS	IGA	JIA	SLE
*N*	8	7	3	2	8
**Humoral response to vaccination** ^a^					
Anti‐S > 250 U/ml	7 (88)	4 (57)	2 (67)	2 (100)	8 (100)
Anti‐S > 100 U/ml	8 (100)	4 (57)	2 (67)	2 (100)	8 (100)
Anti‐S > .8 U/ml	8 (100)	7 (100)	2 (67)	2 (100)	8 (100)
**Cellular response to vaccination**					
Pan‐T‐cell IFNγ response (IU/ml)	.767 (.516, 1.141)	.892 (.606, 1.314)	1.189 (.48, 2.947)	3.002 (1.749, 5.152)	3.97 (2.878, 5.478)
Pan‐T‐cell responder	7 (88)	6 (86)	3 (100)	2 (100)	8 (100)
CD4+ T‐cell IFNγ response (IU/ml)	.239 (.098, .584)	.499 (.327, .761)	.676 (.263, 1.735)	2.579 (1.572, 4.231)	2.38 (1.699, 3.334)
CD4+ T‐cell responder	6 (75)	6 (86)	3 (100)	2 (100)	8 (100)

^a^ Frequency data are given as *N* (%). IFNγ responses are given as the geometric mean (SEM).

Finally, we examined the relationship between vaccine adverse effects and immunogenicity (Table S2). Amongst immunocompromised patients, an adequate pan‐T‐cell response was associated with a decreased prevalence of local side effects (55% vs. 82%, *p* = .018).

The key finding of this study is that within our immunocompromised cohort of young people, there are marked differences in vaccine responses between patients with different underlying conditions. For instance, all LTX patients had anti‐S titers >250 U/ml, compared to only 29% of IGA patients. This was partly associated with the use of immunosuppressive medication, with corticosteroid and anti‐metabolite use being associated with reduced humoral responses, and calcineurin inhibitor use with reduced T‐cell responses, consistent with existing adult data.^3^, ^4^, ^5^, ^6^

However, differing patterns of immunosuppression use do not fully explain the different vaccine responses between disease categories, as there remained significant differences in T‐cell responses even after restricting the analysis to patients on anti‐metabolite monotherapy. This may be due to the immune dysregulation intrinsic to certain conditions. For instance, in IBD, peripheral blood mononuclear cell hyporesponsiveness has been described^7^ as well as therapy‐independent attenuation of responses to various vaccines.^8^

Currently, additional SARS‐CoV‐2 mRNA vaccine doses are prioritised for young patients deemed to have an equivalent level of immunosuppression to solid organ recipients, and various public health bodies have published criteria to define this operationally.^1^, ^2^ In the non‐oncological setting, this would include many young people on various immunosuppressive medications, such as those in our cohort. In this context, our data revealed two main limitations of such an approach. First, given the heterogeneity in vaccine responses, some patient groups who qualify for an additional primary vaccination dose, for example JIA patients, may not benefit significantly, resulting in suboptimal use of vaccines and vaccination capacity. Second, the finding of disease‐specific, immunosuppression‐independent effects on vaccine responses suggest that young patients with mild disease on minimal immunosuppression, for example ulcerative colitis on rectal mesalazine, may also benefit from additional doses of vaccines which they currently do not qualify for. Therefore, further studies defining baseline immunological predictors of vaccine response are urgently required to further inform and optimise vaccination strategies, particularly in this vulnerable patient population.

In conclusion, although immunocompromised young people display attenuated humoral and cellular responses to vaccination in general, there are marked differences in vaccine responses between disease subgroups, not completely explained by immunosuppression used. A more differentiated, evidence‐based framework in determining patient subgroups which will benefit most from additional vaccine doses is required.

## CONFLICTS OF INTEREST

There were no potential conflicts of interest to disclose.

## Supporting information

Supporting Information
