# Supplementary material for: Heterogenous antibody and T‐cell responses to SARS‐CoV‐2 mRNA vaccines among immunocompromised young people
Source: Clin Transl Med. 2023 Jan 19;13(1):e1183. doi: 10.1002/ctm2.1183 (PMC9852384; doi:10.1002/ctm2.1183)
Supplement: Supplementary file 1 — Supporting Information [file CTM2-13-e1183-s001.docx]

**Supplementary Materials**

**Heterogenous antibody and T-cell responses to SARS-CoV-2 mRNA vaccines among** **immunocompromised young people**

**Running title:** COVID vaccines & the immunocompromised young

Liangjian Lu, MRCPCH^#1^, Chang Yien Chan, PhD^#1,2^, Pauline PL Chan-Ng, MRCPCH^#1,2^, Mya Than, MPH^1,2^, Pamela SY Tan, BHealthSci^1^, Lee Kean Lim, BN^1^, Sharon Teo, FRACP^1^, Perry YW Lau, FRCPCH^1,2^, Kar Hui Ng, FRCPCH^1,2^, Elizabeth Y Ang, MMED, MRCPCH^1^, S Venkatesh Karthik, MRCPCH^1^, Marion M Aw, FRCPCH^1,2^, Paul A Tambyah, MD^3^, Hui Kim Yap, MD^1,2^, Bee Wah Lee, MD^2^

^#^These authors contributed equally to this manuscript

^1^ Department of Paediatrics, Khoo Teck Puat- National University Children’s Medical Institute, National University Health System, Singapore

^2^ Department of Paediatrics, Yong Loo Lin School of Medicine, National University of Singapore

^3^ Division of Infectious Diseases, National University Health System and Department of Medicine, Yong Loo Lin School of Medicine, National University of Singapore

Corresponding author:

Liangjian Lu

Department of Paediatrics, Khoo Teck Puat – National University Children’s Medical Institute, National University Health System

Address : Level 12 NUHS Tower Block, 1E Kent Ridge Road, Singapore 119228

Phone : +65 6772 4411

Fax : +65 6779 6401

Email : [david_lu@nuhs.edu.sg](mailto:paeleebw@nus.edu.sg)

**Funding:** This study was funded by Paediatrics Renal Division Fund and Biomedical/Immunology Division Fund. The funding sources played no role in the study design; in the collection, analysis, and interpretation of data; in the writing of the report; and in the decision to submit the paper for publication.

**Conflict of Interest:** There were no potential conflicts of interest to disclose.

Table of Contents

[**Methods** 4](#_Toc116291695)

[Study design and participants 4](#_Toc116291696)

[Clinical data collection 5](#_Toc116291697)

[Laboratory Analysis 5](#_Toc116291698)

[Statistical Analysis 6](#_Toc116291699)

[**Tables** 7](#_Toc116291700)

[Table S1. Tolerance and Variance Inflation Factor (VIF) for variables in multivariable analysis, with and without inclusion of disease categories. 7](#_Toc116291701)

[Table S2. Vaccine adverse effects in relation to vaccine responses within the immunocompromised cohort. 8](#_Toc116291702)

[**Figures** 9](#_Toc116291703)

[Figure S1. Study profile for (a) controls and (b) immunocompromised participants. 9](#_Toc116291704)

[Figure S2. Humoral and cellular responses to vaccination in controls and immunocompromised participants. 10](#_Toc116291705)

[**References** 11](#_Toc116291706)

## Methods

### Study design and participants

Participants were recruited from patients attending the Department of Paediatrics, National University Hospital, between August-November 2021. Initial power calculations revealed that 9 controls and 37 immunocompromised young people would be able to detect a difference between groups with a power of 0.8 for a significance threshold of 0.05, assuming a seroconversion rate of 94% for controls and 50% for immunocompromised participants. However, to ensure a representative cohort of participants across subgroups, recruitment targets were specified as 20 non-immunocompromised controls and 80 to 100 immunocompromised young people.

The inclusion criteria were: (i) between 12-25 years of age at the time of SARS-CoV-2 vaccination and (ii) 3 weeks to 2 months following receipt of 2 doses of either the Pfizer Bnt162b2 or Moderna mRNA-1273 vaccine, received as part of the national vaccination programme. Participants were regarded as immunocompromised if they had end-stage kidney disease (with eGFR <15ml/min/1.73m^2^ or on maintenance dialysis) or were on corticosteroids, anti-metabolites (including methotrexate, mycophenolate mofetil, azathioprine), calcineurin inhibitors or biologics. Participants were excluded if they were (i) oncological patients receiving cytotoxic chemotherapy (as the degree of immunocompromise changes over time), (ii) received Rituximab within 6 months prior to vaccination (not routinely vaccinated at the time of the study in accordance with local guidelines^1^) (iii) had untreated infection, or (iv) known to have previous SARS-CoV-2 infection. Informed consent or assent was obtained from all participants and/or their parents, and ethics approval was obtained from the National Healthcare Group Domain Specific Review Board (2021/00593).

### Clinical data collection

Demographic data, details of clinical diagnosis and immunosuppression were obtained from the clinical records. Data on adverse effects were obtained via self-report using a structured questionnaire, in which participants had to give a dichotomous answer to the following systemic side effects, i.e. fever, fatigue, headache, chills, vomiting, diarrhea, muscle pain, joint pain, chest pain, dyspnea, palpitations, dizziness, rashes, angioedema, abdominal pain or hypotension/syncope, and local side effects, i.e. injection site pain, swelling or erythema.

### Laboratory Analysis

Blood samples were obtained from participants 3 weeks-2 months following completion of the second vaccination dose. Serum levels of antibodies to the spike and nucleocapid proteins of SARS-CoV-2 were measured in NUH Referral Laboratories using the Elecsys® Anti-SARS-CoV-2 S and Anti-SARS-CoV-2 assay respectively on the Cobas analyzer (Roche, Germany). The Elecsys® Anti-SARS-CoV-2 S assay has a detection range of 0.40 to 250 U/ml, and a concentration of >0.80 U/ml is defined as positive for SARS-CoV-2 spike antibody per manufacturer’s instructions. The Elecsys® Anti-SARS-CoV-2 assay is a qualitative assay, with a positive result reflecting previous wild-type infection. All participants tested negative for anti-nucleocapsid antibodies.

Spike protein stimulation was performed using QuantiFERON SARS-CoV-2 Starter Pack (Qiagen, Germany). Whole blood was collected into a sodium-heparin tube and kept at room temperature for not more than 10 hours prior to stimulation. 1ml of blood was subsequently transferred to each SARS-CoV-2 Ag1 and Ag2 tube, which respectively contained peptides targeting CD4+ T-cell and CD4/8+ T-cells (pan-T-cell). Tubes were mixed and incubated at 37^o^C for 18 hours. A negative control tube (unstimulated) and a positive control tube (mitogen stimulated) were also included. Plasma was then collected from each tube by centrifuging for 15 minutes at 3000*g* and stored at -80^o^C for subsequent quantification of interferon gamma (IFNγ) levels using QuantiFERON® ELISA (Qiagen) according to the manufacturer’s instructions.

### Statistical Analysis

T-cell IFNγ production data were log-transformed to reduce skew, and average values are stated as geometric mean (SEM). For the 7 participants in whom IFNγ production was undetectable (3 with undetectable pan-T-cell and CD4+ T-cell responses, 4 with undetectable CD4+ T-cell responses only), a random IFNγ concentration between 0.0001 and 0.001 IU/ml was imputed. Interval variables were compared using Student’s T-test (2 groups) or ANOVA (3 or more groups), with post-hoc testing for the latter performed using Tukey’s test. Proportions were compared using Fisher’s Exact Test (2 groups) or Chi square test (≥3 groups), with post-hoc testing for the latter performed using cellwise residual analysis^2^ while correcting for multiple testing using the Benjamini-Hochberg (BH) procedure. Predictor variables for interval and dichotomous variables were identified using linear and logistic regression respectively. p≤0.05 was regarded as the threshold of statistical significance. Statistical analyses were performed using Microsoft Excel version 2111 (Microsoft, Redmond, WA) and SPSS version 21 (IBM, Armonk, NY). Intersection plot for immunosuppression and disease categories was generated using the UpSetR (version 1.4.0) package.^3^

## Tables

Table S1. Tolerance and Variance Inflation Factor (VIF) for variables in multivariable analysis, with and without inclusion of disease categories. Significant collinearity is associated with Tolerance <0.25 and/or VIF >2.5.

| **Variable** | **Tolerance** | **VIF** | **Tolerance** | **VIF** |
| --- | --- | --- | --- | --- |
| Female | 0.653 | 1.532 | 0.802 | 1.246 |
| Pfizer | 0.672 | 1.488 | 0.779 | 1.284 |
| Age (years) | 0.851 | 1.176 | 0.905 | 1.105 |
| Days between doses | 0.567 | 1.764 | 0.667 | 1.499 |
| Days from dose 2 | 0.868 | 1.152 | 0.973 | 1.028 |
| Corticosteroids | 0.359 | 2.783 | 0.605 | 1.652 |
| Anti-metabolites | 0.336 | 2.976 | 0.631 | 1.585 |
| Calcineurin inhibitors | 0.294 | 3.398 | 0.685 | 1.460 |
| Biologics | 0.351 | 2.851 | 0.800 | 1.250 |
| IBD | 0.289 | 3.455 | NA | NA |
| KTX | 0.362 | 2.762 | NA | NA |
| LTX | 0.289 | 3.466 | NA | NA |
| INS | 0.320 | 3.122 | NA | NA |
| ESKD | 0.239 | 4.177 | NA | NA |
| IGA | 0.409 | 2.443 | NA | NA |
| JIA | 0.408 | 2.451 | NA | NA |
| SLE | 0.262 | 3.816 | NA | NA |
| UVI | 0.567 | 1.763 | NA | NA |

IBD: Inflammatory bowel disease; KTX: Kidney transplant; LTX: Liver Transplant; INS: Idiopathic nephrotic syndrome; ESKD: End-stage kidney disease; IGA: IgA nephropathy and IgA vasculitis; JIA: Juvenile idiopathic arthritis; SLE: Systemic lupus erythematosus; UVI: Idiopathic uveitis; NA: not applicable

### Table S2. Vaccine adverse effects in relation to vaccine responses within the immunocompromised cohort.

|  | **Controls** | **Immunocompromised** | | | | | | | | | |
| --- | --- | --- | --- | --- | --- | --- | --- | --- | --- | --- | --- |
|  |  | All | Anti-S <250 U/ml | Anti-S >250 U/ml | p-value | Pan-T-cell  Non-responder | Pan-T-cell responder | p-value | CD4+ T-cell Non-responder | CD4+ T-cell responder | p-value |
| N | 20 | 93 | 25 | 68 |  | 27 | 66 |  | 21 | 72 |  |
| Fever (N, %) | 8 (40) | 29 (31) | 4(16) | 25 (37) | 0.077 | 6 (22) | 23 (35) | 0.325 | 4 (19) | 25 (35) | 0.195 |
| Systemic side effects (N, %) | 16 (80) | 56 (60) | 12 (48) | 44 (65) | 0.159 | 16 (59) | 40 (61) | >0.999 | 10 (48) | 46 (64) | 0.211 |
| Local side effects (N, %) | 12 (60) | 58 (62) | 17(68) | 41 (60) | 0.631 | 22 (82) | 36 (55) | 0.018* | 15 (71) | 43 (60) | 0.444 |

* refers to p<0.05.

## Figures

**Figure S1**


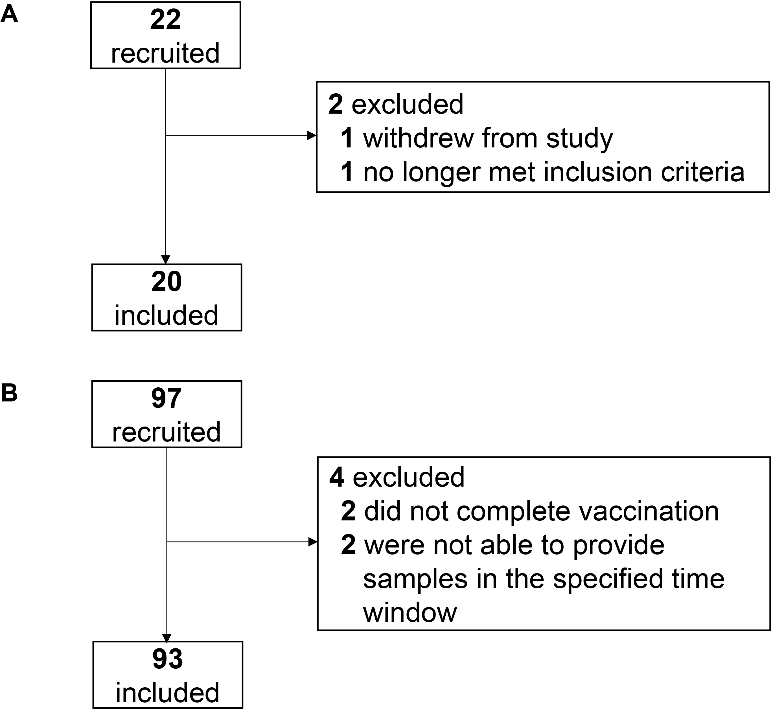


### Figure S1. Study profile for (a) controls and (b) immunocompromised participants.

**Figure S2**

**
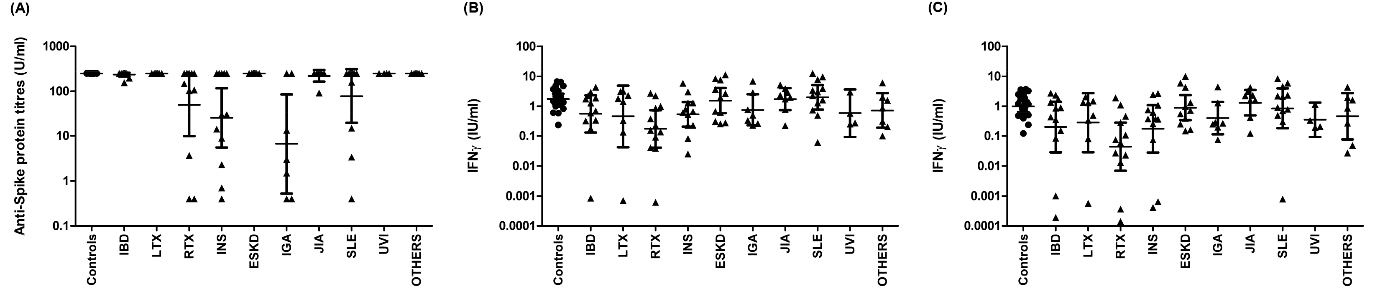
**

Figure S2. Humoral and cellular responses to vaccination in controls and immunocompromised participants. (A) Anti-Spike protein titres, (B) Pan-T-cell IFNγ, and (C) CD4+ T-cell IFNγ responses in controls and immunocompromised participants by disease group.

## References

1. Santosa A, Xu C, Arkachaisri T, et al. Recommendations for COVID-19 vaccination in people with rheumatic disease: Developed by the Singapore Chapter of Rheumatologists. *Int J Rheum Dis*. Jun 2021;24(6):746-757. doi:10.1111/1756-185X.14107

2. Sharpe D. Chi-Square Test is Statistically Significant: Now What? *Practical Assessment, Research, and Evaluation*. April 2015 2015;20(2015)(Article 8); doi:https://doi.org/10.7275/tbfa-x148

3. Conway JR, Lex A, Gehlenborg N. UpSetR: an R package for the visualization of intersecting sets and their properties. *Bioinformatics (Oxford, England)*. Sep 15 2017;33(18):2938-2940. doi:10.1093/bioinformatics/btx364
